# Study on the Uniaxial Compression Constitutive Relationship of Wood Reinforced with Fiber-Reinforced Polymer

**DOI:** 10.3390/polym17081119

**Published:** 2025-04-20

**Authors:** Hao Chen, Zihui Zhang, Zhihui Wang, Yongcheng Ji

**Affiliations:** 1 School of Civil Engineering and Transportation, Northeast Forestry University, Harbin 150040, China; 17367998217@163.com; 2 Aulin College, Northeast Forestry University, Harbin 150040, China; polly_zhang_nefu@imtcoeu.org (Z.Z.); wang_zhihui_nefu@tdaio.org (Z.W.)

**Keywords:** BFRP, CFRP, mechanical properties, finite element model, constitutive model

## Abstract

Fiber-reinforced polymer (FRP) composites demonstrate significant advantages in the reinforcement of timber structures, with basalt fiber-reinforced polymer (BFRP) and carbon fiber-reinforced polymer (CFRP) exhibiting distinct characteristics. This study systematically compares the mechanical performance differences between BFRP- and CFRP-reinforced Northeast larch timber columns. Uniaxial compression tests focused on the mechanical responses under different reinforcement conditions along the grain direction. The results indicate that BFRP-reinforced specimens exhibit superior cost-effectiveness, enhanced ductility, and improved damage tolerance, whereas CFRP-reinforced specimens demonstrate higher stiffness and ultimate load-bearing capacity. A damage constitutive model, developed based on Poisson distribution theory, accurately describes the damage evolution process of fully FRP-reinforced Northeast larch timber columns. Numerical simulations show excellent agreement with experimental results. The study provides critical guidance for FRP material selection and reinforcement strategies in timber structure engineering: BFRP is more suitable for general applications prioritizing cost efficiency and ductility, while CFRP is better suited for special structures requiring higher load-bearing capacity. Finite element models of CFRP- and BFRP-reinforced timber specimens under axial compression were established using ABAQUS 2020 software, with simulation results closely matching experimental data. The proposed constitutive model and finite element analysis method offer a reliable tool for predicting the mechanical behavior of FRP-wood composite structures.

## 1. Introduction

In recent years, China has promulgated a series of policies and regulations to promote the transformation and upgrading of the construction industry. The 13th Five-Year Plan mentions the need to develop applicable, economic, green, and aesthetically pleasing buildings and advocates and promotes assembly buildings. In 2019, the National Development and Reform Commission issued the Overall Programme for Green Living Creation Action, which mentioned that by 2022, the green living creation action will achieve significant results, and the proportion of green building area in new cities and towns will reach 60%. The future of China’s construction industry will increasingly focus on the core concepts of green materials, green construction, and green life. Wood is a natural and sustainable material characterized by its lightweight, high strength, and excellent processing performance. At the same time, it is one of the world’s most environmentally friendly building materials due to its low energy consumption and low emissions [1].

Due to natural environmental factors such as seismic hazards, prolonged wind, and sand erosion, chemical corrosion damage, as well as wood’s inherent vulnerabilities to decay, insect infestation, and natural defects including knots, drying cracks, fissures, and creep, timber structures inevitably sustain varying degrees of damage over time [2]. Fiber Reinforced Polymer (FRP) materials have been used increasingly in wood structure reinforcement in recent years due to the advantages of being lightweight and high strength, having good durability, good fatigue resistance, and designability [3]. The research on reinforcing timber structures using GFRP (Glass Fiber-Reinforced Polymer) composites began in the 1960s. Wangaard [4] pioneered this field by proposing and experimentally validating the reinforcement of timber beams with glass fibers. Spaun F.D. [5] and others chose to reinforce timber with glass fiber to study the mechanical properties after reinforcement. F. Taheri [6] et al. carried out a numerical computational study on FRP-reinforced axially compressed glued laminated timber columns by investigating the effect of experimental parameters such as the length to slenderness ratio of the columns, boundary conditions, and FRP reinforcement method on the mechanical properties of the reinforced columns and analyzing them in terms of their economy. It was found that the most ideal reinforcement method is to use FRP to wrap the middle part of the column, and the wrapped area is 33.3% of the whole column. This study also provides a valuable reference for the application of practical engineering. Jinsong Shao [7] et al., based on anisotropic elasticity mechanics, established the calculation formula of yield load and yield strain of reinforced wooden columns according to the Tsai Wu strength criterion and conducted the axial compressive performance test of 15 FRP-reinforced wooden columns, to explore the working mechanism and damage modes of the specimens after being loaded in detail.

By pasting FRP cloth on the tension surface of square timber, FRP material’s high specific strength and good corrosion resistance are utilized to enhance square timber’s load-carrying capacity and stiffness. In this paper, FRP-reinforced northeastern larch timber is investigated in a one-way smooth grain compression test to provide a reference in terms of the mechanical properties of FRP-reinforced wood structures.

Wood is a typical anisotropic material, and its mechanical properties show different characteristics in different directions, especially in the nonlinear stress stage. The difference between the stress behavior and damage mode in different directions is significant, which is an important reason for the complexity of the stress performance of wood structures and their components. Mastering the nonlinear force behavior of wood is conducive to understanding the force mechanism and force performance of the wood structures and their components. The force analysis of wood structures and their components requires selecting appropriate wood constitutive models, especially when numerical analysis methods are becoming the mainstream analysis means, and their force analyses’ accuracy, efficiency, and reliability are constantly improving. There is an urgent need to establish constitutive models that can better reflect wood’s anisotropic and nonlinear force characteristics [8,9].

The constitutive behavior of wood is characterized through relationships that can be modeled using two fundamental approaches empirical models derived from experimental data fitting, categorized by loading conditions (monotonic/cyclic) and rate effects static/dynamic and theoretical frameworks including elastic, elasto-plastic, and elasto-plastic damage formulations based on continuum mechanics principles [10]. This systematic classification enables comprehensive simulation of wood’s nonlinear response across all loading stages.

The empirical principal model for wood is based on analyzing the characteristics of its experimentalstress-strain relationship curves in each grain direction and selecting a suitable function, which is fitted by the least squares method to determine the stresses, which can be expressed: σ=f(ε) ,where it is dominated by forms such as linear, polynomial, power functions or using a combination of segments of the above functions [11,12,13,14,15].

Scholars have established uniaxial eigenstructure models of wood using different functional forms for the stress characteristics of wood in different grain directions, including the monotonic stress eigenstructure model, repetitive stress eigenstructure model, and dynamic rate eigenstructure model [16,17]. The elastic plastic constitutive model of wood is based on the theoretical framework of plastic mechanics of metallic and various non-metallic materials (e.g., composites, rocks, soils, and concrete) and is established by considering wood’s unique nonlinear force behavior. Two main types of methods are used to establish elastic plastic intrinsic models in plasticity mechanics theory, namely, the incremental method (flow theory) and the allometric method (deformation theory). The former cannot consider the effect of stress paths. At the same time, the latter can reflect the historical correlation of stresses and is commonly used in elastoplastic constitutive modeling of various materials [18].

Damage mechanics mainly studies the process of macroscopic property deterioration and damage caused by initial defects under external loading of materials [19], which can reflect the developmental evolution of initial defects. The damage constitutive model is established based on damage mechanics theory through the damage variables (can be defined as a single scalar, bivariate, multi-scalar, tensor, and other forms) to describe the material beyond the peak strength of the strain softening, unloading stiffness degradation, and other nonlinear behaviors, and plasticity theory can reflect the plasticity of the material at the same time and the mechanism of the damage [20]. Therefore, the damage eigenmodel is divided into the elastic damage eigenmodel and the elastic plastic damage eigenmodel, depending on whether it reflects the plastic mechanism of the material.

Given the above research, this study adopts the Poisson distribution probability density function to establish the wood damage eigenmodes. This function determines the control parameters in the damage eigenmodes combined with the deformation and damage curve, establishing the damage eigenmodes suitable for the wood damage of northeastern larch.

## 2. Specimen Preparation and Experimental Tests

### 2.1. Raw Materials and Raw Material Properties

The timber selected for this test is northeastern larch, and the specimens are all taken from the Yichun City Forestry Farm in Heilongjiang Province. According to the Standard for Design of Timber Structures (GB/T 50005-2017) [21], the specimens were prepared with the size of 100 mm × 100 mm × 400 mm prismatic specimens. The moisture content of the wood specimens was 12.3%, and the density was 0.59 g/cm^3^, and the material parameters are shown in Table 1.

**Figure 1:**
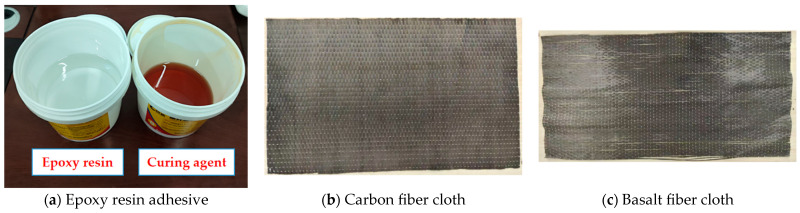
Epoxy Resin and FRP.

This experimental study employed two high-performance fiber-reinforced polymer (FRP) fabrics manufactured by Zhongfu Shenying Carbon Fiber Co., Ltd. (Xining, China): carbon fiber-reinforced polymer (CFRP) fabric (Figure 1b) and basalt fiber-reinforced polymer (BFRP) fabric (Figure 1c), with their principal mechanical properties detailed in Table 2 and Table 3, respectively. The epoxy adhesive system, consisting of Component A (semi-transparent milky epoxy resin) and Component B (dark-red curing agent) mixed at a 2:1 mass ratio (Figure 1a) was also supplied by the same manufacturer. Quality control tests confirmed that all technical parameters of the structural adhesive (Table 4) strictly comply with the Technical Code for Safety Appraisal of Engineering Structure Strengthening Materials (GB 50728-2011) [22]. The integrated strengthening system, comprising CFRP/BFRP fabrics and the compatible epoxy matrix, fully satisfies the safety requirements stipulated in Chinese national standards for structural reinforcement materials.

The experimentally measured mechanical properties of the materials are summarized as follows: the CFRP fabric exhibited a tensile strength of 3820 MPa and an elastic modulus of 238 GPa. In contrast, the BFRP fabric demonstrated 2150 MPa tensile strength and 89 GPa elastic modulus, as determined through standardized testing per ASTM D3039. The epoxy adhesive system achieved a tensile strength of 45.6 MPa and shear strength of 18.2 MPa when cured at 23 ± 2 °C for 7 days, with all values exceeding the minimum requirements specified in GB 50728-2011 [21] for structural strengthening applications.

### 2.2. Fabrication and Maintenance of Specimens

**Figure 2:**
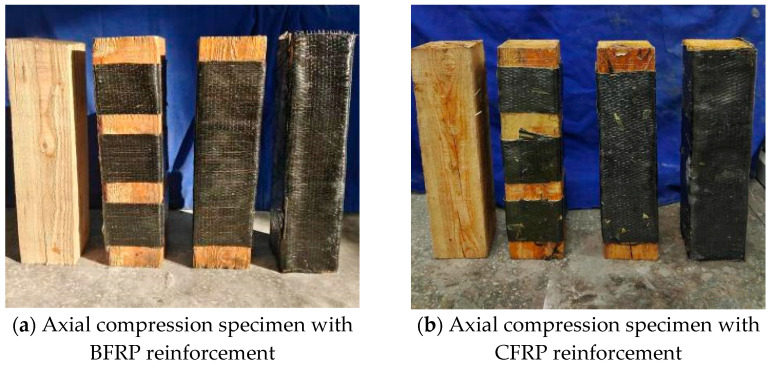
Square wood axial compression specimen.

**Figure 3:**
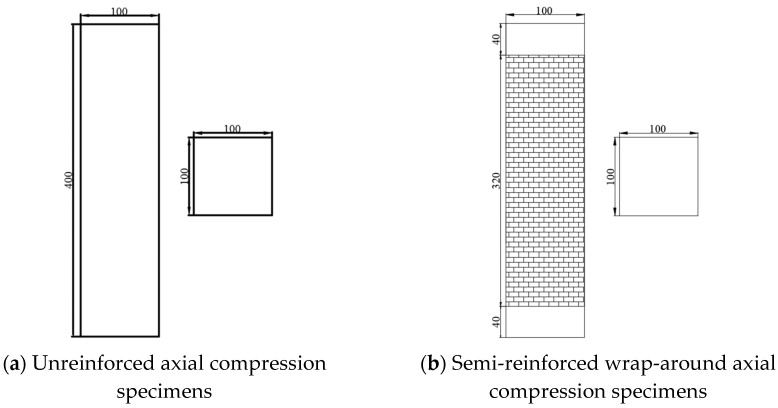

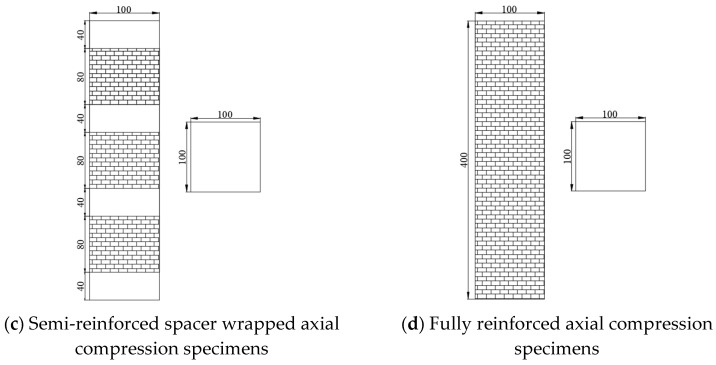
Dimensions of axial compression specimen (unit: mm).

Twenty-one square timber specimens (100 × 100 × 400 mm) were fabricated for axial compression testing, with fiber reinforcement type and configuration as primary variables. Four distinct CFRP adhesion schemes were implemented: unreinforced control (0%), spiral-wrapped (60% coverage via 320 mm-wide strip), spaced-strip (80% coverage using three 80 mm-wide strips with 40 mm spacing), and full wrap (100% coverage with 400 mm-wide CFRP). All reinforced specimens received epoxy end-coating (Zhongfu Shenying E-45, 2:1 resin/hardener, Xining, China) and were cured for 7+ days at 23 ± 2 °C. Critical design considerations included (1) standardized 100 mm lap lengths (25% perimeter) to ensure CFRP rupture failure mode [23] and (2) vacuum bagging (0.08 MPa) for void elimination. Identical procedures were followed for BFRP specimens. Specimen geometries and reinforcement configurations are detailed in Figure 2 and Figure 3 (dimensional tolerance: ±0.5 mm).

According to the difference in reinforcement materials and reinforcement types, the square wood axial pressure specimens were grouped and numbered, with three specimens in each group, and 12 axial pressure specimens, each of square wood reinforced with carbon fiber cloth and basalt fiber cloth, making a total of 24 specimens. Among them, “ZY” means axial pressure, “ZY0” means unreinforced square wood specimen, “ZYC” means reinforced square wood specimen material is carbon fiber cloth, “ZYB” indicates that the reinforced square wood specimen material is basalt fiber cloth, and at the same time, according to the rate of reinforcing material pasting area of the same material, three types of reinforcement divided into 60%, 80%, and 100% correspond to the half-reinforcement spacing parcel, half-reinforcement wrapping parcel, and full-reinforcement axial compression specimen, respectively. Specific numbering information is shown in Table 5.

### 2.3. Loading Programme

**Figure 4:**
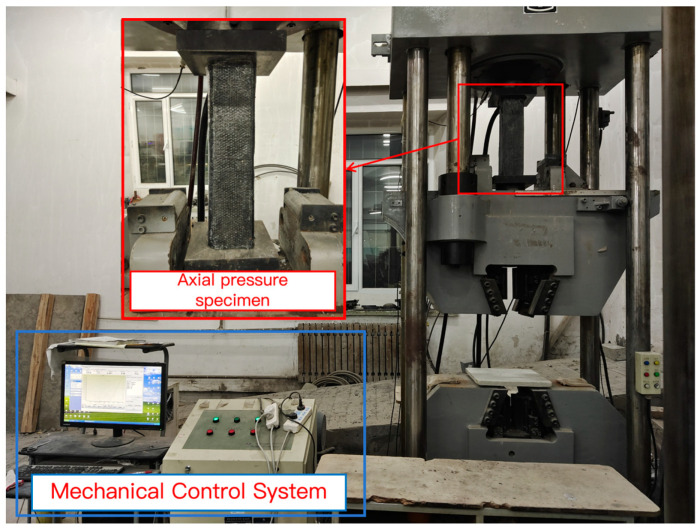
Axial pressure test.

This test was conducted with 21 groups of square wood specimens under different reinforcement conditions as the research object, and the YAW-500F microcomputer-controlled electro-hydraulic servo pressure testing machine (Figure 4) was used as the core loading equipment. The test system consists of two modules: mechanical control and data acquisition. The experimental process of obtaining the load–displacement curve consists of mechanical control and data acquisition systems. The former is responsible for loading the specimen by controlling the YAM-5000F servo-hydraulic pressure testing machine (Jinan Testing Equipment IE Corporation, Jinan, China) and collecting the pressure value on the specimen. The latter is connected to the computer through a static data acquisition instrument. The test force and displacement are collected simultaneously, and the displacement before and after deformation is collected. Finally, the data collected by the computer is processed and analyzed.

This test strictly follows the specification requirements of the “Standard for Test Methods for Timber Structures” (GB/T 50329-2012) [24] and ensures the accuracy of the axial compression test through three-stage control: firstly, the geometric alignment is conducted to make the bearing surface of the specimen accurately aligned with the press plate, and the deviation is controlled within ±0.5 mm; Second, preloading (less than or equal to 20% of the estimated failure load) is implemented to eliminate assembly clearances. The formal loading adopts the displacement control mode (0.5 mm/min). The curve is continuously monitored during the loading process, and the test is terminated when the load drops to 70% of the peak load. Three parallel specimens were set up in each group, and the final data were averaged to eliminate discreteness. The whole test process is completed within 6–10 min, which meets the loading rate requirements specified in the standard and accurately captures the specimen’s nonlinear response characteristics.

## 3. Test Results and Analyses

### 3.1. Axial Pressure Test Results

The experimental results demonstrate that externally bonded carbon fiber-reinforced polymer (CFRP) sheets and basalt fiber-reinforced polymer (BFRP) sheets significantly enhance square timber columns’ ultimate bearing capacity and stiffness. Specifically, the ultimate bearing capacity of CFRP-strengthened columns increased by 8.57% to 16.68%, while that of BFRP-strengthened columns increased by 7.73% to 11.58%. Under the same fiber sheet material conditions, the average ultimate bearing capacity of the specimens significantly improved with increasing fiber-strengthened area ratio. However, when the fiber-strengthened area ratios reached 60% and 80%, the difference in the strengthening effects between the two methods on the ultimate bearing capacity was insignificant. Additionally, the ductility coefficient of the strengthened specimens increased by 11.87% to 15.68%, and the ductility coefficient rose with the increase in fiber sheet bonding area. Notably, CFRP exhibited superior performance over BFRP in bearing capacity and ductility enhancement, which may be attributed to CFRP’s higher elastic modulus and tensile strength.

For the accurate evaluation of ductility performance in FRP-strengthened specimens, this study employed the displacement ductility coefficient calculation (ductility coefficient = failure displacement/yield displacement) proposed in Reference [25], where the yield load was taken as 85% of the ultimate load with its corresponding displacement defined as the yield displacement, while the failure state was determined when unloading to 85% of the ultimate load with the corresponding displacement regarded as the failure displacement. All specimens’ ductility coefficients were obtained through this calculation method, and their mechanical performance parameters and calculation results are presented in Table 6.

### 3.2. Test Damage Phenomena

**Figure 5:**
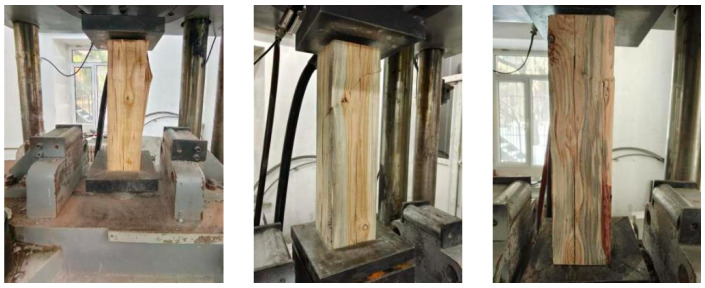
Damage pattern of unreinforced axial compression specimen.

The axial compression failure of unreinforced timber columns exhibited typical brittle failure characteristics characterized by a three-stage evolution mechanism: during the initial loading stage, no significant deformation was observed on the specimen surface while the curve demonstrated linear elastic response; when approaching the ultimate load range (255–275.5 kN), internal fiber micro-buckling occurred accompanied by intermittent cracking sounds, with longitudinal micro-cracks parallel to the wood grain appearing on the surface; at peak load, sudden instability occurred with stress concentration nuclei forming at initial defects (e.g., natural knots or micro-cracks), triggering rapid crack propagation along weakest paths—specifically, specimen ZY0-1 showed shear failure induced by local bulging in the upper-right section, ZY0-2 displayed tensile failure with 45° diagonal splitting, and ZY0-3 developed transverse fractures perpendicular to loading direction, with the entire failure process demonstrating abrupt characteristics where residual capacity abruptly dropped below 60% of peak load after main crack penetration while maintaining structural integrity in remaining sections, as illustrated in Figure 5.

**Figure 6:**
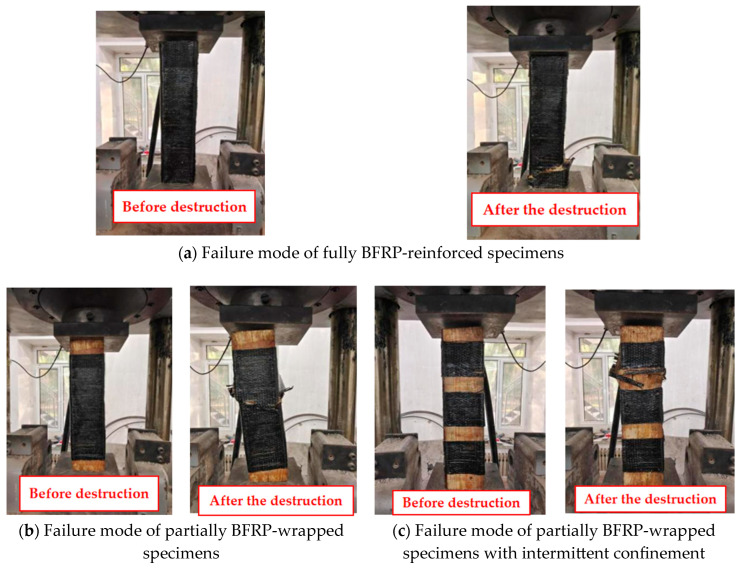
Failure mode of BFRP-strengthened specimens under axial compression.

As shown in Figure 6a, specimens ZYB100-1 to ZYB100-3 represent timber columns fully reinforced with BFRP (Basalt Fiber Reinforced Polymer), demonstrating a multi-stage progressive failure mechanism. During the initial loading phase, adhesive layer imperfections induced interfacial debonding, with audible cracking sounds in ZYB100-3 originating from localized adhesive crushing at the ends. As the load increased, the constraining effect of the fiber reinforcement caused either mid-column bulging deformation (ZYB100-3) or end cracking (ZYB100-1/2). Upon approaching peak load conditions (294.56–299.91 kN), a sudden fracture occurred in the BFRP layer accompanied by adhesive failure-induced horizontal displacement (particularly evident in ZYB100-2). This reinforcement system effectively transformed the failure mode from the characteristic brittle splitting of unreinforced columns to a more ductile shear failure pattern, ultimately manifesting as diagonal splitting cracks at the base section.

As illustrated in Figure 6b, specimens ZYB80-1 to ZYB80-3 represent timber columns partially reinforced with a 320 mm-long BFRP strip at their mid-section. These partially reinforced columns exhibited a coupled “constraint failure-local buckling” failure mechanism: During initial loading, the circumferential constraint provided by the BFRP effectively restrained lateral deformation of the wood, maintaining the specimens in a linear elastic state. When the load exceeded a critical threshold of approximately 300 kN, local buckling first occurred in the unreinforced wood segments, accompanied by acoustic emission signals indicating interfacial debonding between the fiber reinforcement and wood substrate. With further load increase, the mid-reinforced section experienced BFRP rupture due to insufficient lateral confinement, resulting in a sudden effective constraint length. The final failure manifested as fiber reinforcement fracture at the column mid-height, followed by abrupt loss of compressive load-bearing capacity and structural collapse of the timber section.

Figure 6c shows that the BFRP-intermittently reinforced timber columns exhibited a “multi-stage, asymmetric” failure evolution. During initial loading, the discretely distributed BFRP strips created discontinuous confinement zones, maintaining the global stability of the specimen. Upon exceeding the critical load, local micro-buckling occurred in unreinforced wood segments due to insufficient lateral confinement. Approaching peak load (281.46–295.7 kN), stress concentration caused brittle fracture of BFRP strips in reinforced zones, leading to a sudden confinement width. Subsequently, second-order effects induced bending deformation in the upper column section. The final failure pattern manifested as alternating wood crushing between reinforcement strips and BFRP fracture, demonstrating the progressive nature of this hybrid failure mechanism.

**Figure 7:**
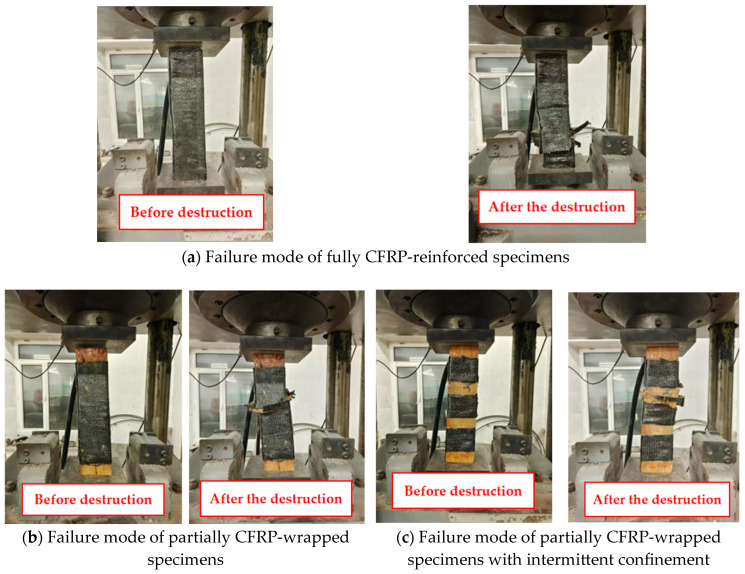
Failure mode of CFRP-strengthened specimens under axial compression.

Figure 7 illustrates the failure characteristics of CFRP-strengthened specimens, demonstrating failure phenomena and mechanisms similar to those of BFRP-reinforced specimens but exhibiting more pronounced plastic development characteristics. The specimens appeared intact during the initial loading stage (≤60% of ultimate load). When the load reached 80% of ultimate capacity, longitudinal micro-cracks occurred on the CFRP surface accompanied by noticeable transverse expansion. Frequent fiber rupture sounds occurred when approaching peak load, followed by ultimate failure. Unlike the sudden failure of unstrengthened specimens, CFRP-reinforced specimens displayed progressive post-peak failure characteristics: reduced load degradation rate and multi-stage fiber rupture patterns. Initial interfacial debonding occurred at weak zones (e.g., lap splice ends), followed by gradual fiber breakage, forming through-thickness cracks (3–5 mm width) while maintaining structural continuity with a residual capacity exceeding 45% of ultimate load. This failure mode confirms CFRP reinforcement effectively mitigates wood’s brittle behavior through fiber bridging effects that retard crack propagation, enhancing ductility and damage tolerance. Comparative mechanical parameters between strengthened and unstrengthened specimens are presented in Table 6.

### 3.3. Load–Displacement Curve Analysis

**Figure 8:**
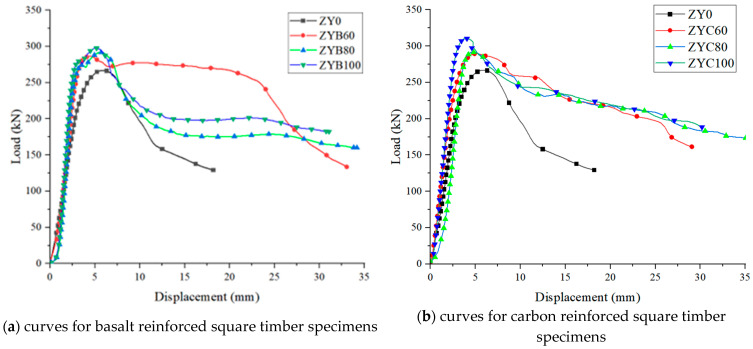
Curves of each group of reinforced specimens.

This study reveals the mechanical behavior characteristics of fiber-reinforced timber through a comparative analysis of curves between BFRP- and CFRP-strengthened square timber specimens, as shown in Figure 8. Test results demonstrate that all strengthened specimens exhibited significantly higher stiffness (curve slope) in the elastic stage compared to unstrengthened specimens (ZY0), with CFRP specimens (ZYC100) showing an 82% increase in initial stiffness and BFRP specimens (ZYB100) achieving 65% improvement, confirming fiber composites effectively enhance deformation resistance.

During the failure phase, unstrengthened specimens displayed typical brittle behavior with 90% load drop at 5.2 mm displacement, whereas strengthened specimens exhibited pronounced ductility: CFRP specimens demonstrated gradual post-peak degradation, with ZYC100 maintaining 75% residual capacity at 25 mm displacement and reaching 32.5 mm ultimate displacement (6.2 times that of control specimens). BFRP specimens showed intermediate performance (ZYB100: 28.7 mm ultimate displacement).

This divergence stems from material properties: CFRP’s superior fracture toughness and elastic modulus facilitated sequential fiber-bundle rupture, creating multi-stage plateau regions in the curve. BFRP’s lower modulus led to continuous degradation, reflecting progressive fiber-matrix interface failure. Energy dissipation analysis showed CFRP specimens (ZYC100: 12.5 kJ, 380% increase) outperformed BFRP (ZYB100: 8.7 kJ, 260% increase) in cumulative energy absorption. The data conclusively prove that FRP strengthening enhances load-bearing capacity (ZYC100: 156% peak load increase) and, more importantly, significantly transforms failure modes to improve ductility and ultimate compressive performance. While CFRP demonstrates superior comprehensive performance, BFRP offers cost advantages for engineering applications.

Through comparative analysis of the curves of four specimen groups (ZYB60, ZYB80, ZYC60, ZYC80) in Figure 8, the following conclusions can be drawn:

All strengthened specimens exhibited typical two-phase mechanical behavior. Load and displacement maintained a linear relationship during the elastic phase (Phase I). Specimens ZYC60 and ZYC80 demonstrated elastic behavior up to approximately 240 kN and 260 kN, respectively, representing 83.2–84.1% of their ultimate loads, while ZYB60 and ZYB80 reached 210 kN and 220 kN, accounting for 82.4–82.9%. Compared to unstrengthened specimens, CFRP strengthening increased the elastic load range by 22.7%, whereas BFRP achieved a 13.6% improvement.

In the plastic phase (Phase II), CFRP-strengthened specimens (ZYC60/ZYC80) showed more gradual stiffness degradation, with significantly superior plastic deformation capacity to BFRP specimens. The curves of BFRP specimens (ZYB60/ZYB80) exhibited intermediate descent rates between CFRP and unstrengthened specimens. Partially strengthened specimens (ZYB60/ZYC60) demonstrated lower plastic deformation capacity than fully wrapped specimens.

Experimental data and curves indicate that CFRP outperforms BFRP in enhancing elastic load range and plastic deformation capacity. When the reinforcement ratio increased from 60% to 80%, CFRP specimens showed more significant performance improvement (15%) than BFRP specimens (8%). The elastic phase proportion (79.9–84.1%) was less affected by reinforcement material and primarily determined by the inherent characteristics of the timber itself.

Wood can be simplified as an orthotropic material. The relationship is assumed to be orthotropic in the elastic stage and isotropic in the plastic stage. The assumption of orthotropic behavior in the elastic stage and isotropic behavior in the plastic stage for wood is grounded in its inherent microstructure and observed mechanical response under loading. The elastic modulus was determined through material property tests, with the average compressive modulus parallel to grain identified as 10,495.99 MPa, as shown in Table 7.

### 3.4. Ultimate Bearing Capacity Analysis

According to the Standard for Design of Timber Structures (GB/T 50005-2017) [21], the verification of axially compressed members shall be categorized based on specific conditions. Strength criteria govern the ultimate bearing capacity for a short column of Northeast China larch with cross-sectional dimensions of 100 mm × 100 mm × 400 mm (slenderness ratio λ = 13.86 < 17). When evaluating the compressive capacity based on strength, the following formula shall be applied:

(1) $$N=A_{n}f_{cd}$$

In the formula, $f_{cd}$: Design value of compressive strength parallel to grain (N/mm²); N is the design axial compressive force (N); $A_{n}$: Net cross-sectional area of the compression member (mm^2^), which shall be determined in accordance with Article 5.1.3 of this standard.

In this study, the average compressive strength parallel to grain of China larch obtained from material tests was 35.67 MPa. According to the Standard for Design of Timber Structures (GB/T 50005-2017) [21], the partial coefficient (γ) for timber typically ranges from 1.2 to 1.5. Considering the actual conditions of this research, a value of 1.3 was adopted for γ. The design value is calculated using the following formula:

(2) $$f_{cd}=\frac{f_{c}}{\gamma }$$

The calculated design compressive strength is determined to be 27.44 MPa. With a net cross-sectional area of 10,000 mm^2^ and a moisture content adjustment factor 1.0, the theoretical bearing capacity is computed as 274.4 kN, showing a slight 4.1% overestimation compared to the experimental mean value of 266.75 kN. This minor deviation, which falls within acceptable engineering tolerances, primarily stems from unaccounted factors in the theoretical model, including natural timber defects (knots, cracks) and test boundary condition effects, demonstrating the complementary relationship between analytical predictions and experimental validation in timber structural analysis.

The experimental results demonstrate that the axial compression specimens strengthened with CFRP and BFRP fabrics exhibited significant improvements in ultimate bearing capacity compared to unstrengthened specimens, with enhancement ratios ranging from 7.73% to 16.70%. Specifically, the CFRP-strengthened specimens (ZYC60, ZYC80, ZYC100) showed average ultimate capacity increases of 8.57%, 9.41%, and 16.70%, respectively, while the BFRP-strengthened specimens (ZYB60, ZYB80, ZYB100) achieved improvements of 7.73%, 9.38%, and 11.58%, respectively. CFRP generally outperformed BFRP in strengthening effectiveness, particularly under full wrapping conditions (100% area ratio), where CFRP specimens demonstrated a significantly higher capacity enhancement (16.70%) compared to BFRP specimens (11.58%).

**Figure 9:**
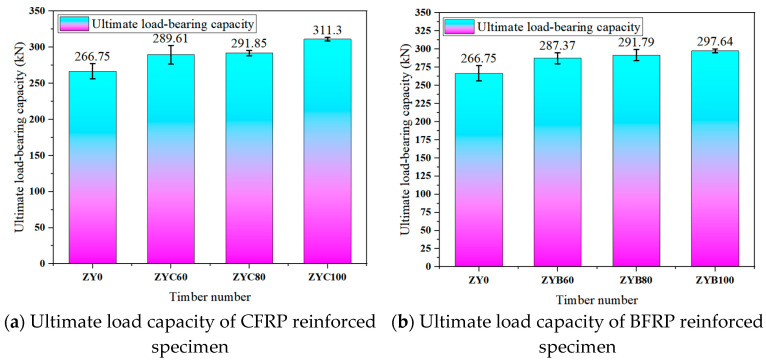
Ultimate bearing capacity of axial compression specimen.

The test results revealed distinct performance characteristics among different strengthening configurations: specimens enhanced ultimate bearing capacity most substantially. In contrast, those with spaced wrapping (80% coverage ratio) exhibited marginally better performance among partially strengthened specimens than spiral-wrapped specimens (60% coverage ratio). Notably, the study demonstrated that for a given fiber reinforcement material, the specific arrangement pattern had negligible influence compared to the decisive role of the bonded area ratio in determining strengthening effectiveness. These findings provide critical design guidelines: CFRP full wrapping delivers optimal load-bearing improvement (16.7% increase), whereas BFRP spaced wrapping (80% coverage) offers the most cost-efficient solution, achieving 90% of CFRP’s performance at 60–70% of the material cost, which a key consideration for practical engineering applications as quantitatively illustrated in Figure 9.

## 4. Constitutive Model of Timber Under Compression

The timber constitutive model is the basis for stress analysis of wood structures. Studies have shown that when establishing the constitutive model, it is necessary to consider the material properties and the force state to modify the shape and scale parameters, such as the concrete damage model [26], the saturated wind-logged soil damage hardening model [27], and the rock damage softening model [28].

It is shown that the parameter solution method of Weibull distribution is used for concrete, rock, and other materials with minor computational errors but is used for wood with significant computational errors; the normal distribution is not suitable for the construction of wood constitutive model; the lognormal distribution can be used for the construction of tensile segments in wood constitutive model. Compared with other distribution functions, Poisson distribution is more suitable for establishing the damage constitutive model of wood [29]. Therefore, this experiment adopts the Poisson distribution probability density function to establish the damage constitutive model of wood, combined with the geometric conditions of the deformation damage curve of wood in the direction of the grain, to give the method of determining the control parameters in the damage constitutive model, and based on the test of Northeast larch timber, to compare the results of the test with the results of the theoretical model, to validate the feasibility of the model.

Based on Lemaitre’s theory of strain equivalence [30], the damage eigenrelationship equation for wood can be formulated as follows:

(3) σ=Eε(1—D(ε)) 

where E is the initial modulus of elasticity, *ε* is the strain, σ is the stress, and D(ε) is the damage variable characterized by the strain.

If the wood is discrete and regarded as a collection of countless microelements, the probability density function, f(ε), can be used to represent the statistical distribution law of the strength of wood microelements. Under the action of external loading, wood will be damaged and deformed, and the damage, D, is the macroscopic manifestation of the cumulative effect of the damage of the microelement body, which can be expressed as follows:

(4) $$D(\varepsilon )=\int_{0}^{\varepsilon }f(t)dt$$

where D is the load damage variable.

The probability density of the Poisson distribution as a function of the damage distribution is a

(5) $$f(\varepsilon )=\frac{k}{m}{\left(\frac{\varepsilon }{m}\right)}^{2k-1}\exp\left[-{\left(\frac{\varepsilon }{m}\right)}^{k}\right]$$

where *m* is the scale parameter and k is the shape parameter.

Bringing Equation (5) into Equation (4) yields an expression for its wood damage variable.

(6) $$D(\varepsilon )=\int_{0}^{\varepsilon }f(t)dt=1-\left[1+{\left(\frac{\varepsilon }{m}\right)}^{k}\right]\exp\left[-{\left(\frac{\varepsilon }{m}\right)}^{k}\right]$$

The peak point of the curve for wood damage was used as the boundary condition as follows:

$$①\;\varepsilon =\varepsilon_{pk},\;\sigma =\sigma_{pk};\;②\;\varepsilon =\varepsilon_{pk}.\;\frac{d\sigma }{d\varepsilon }=0$$

Let ε=m, the stress σ is equal to $\frac{2Em}{e}$, and the curve is constant over the point (m, $\frac{2Em}{e}$), although the parameters are different. Assign a value to k to obtain the corresponding m. Bring k and m into the constitutive equation to iterate the stress values under different parameters:

(7) $$D(\varepsilon )=\int_{0}^{\varepsilon }f(t)dt=1-\left[1+{\left(\frac{\varepsilon }{m}\right)}^{k}\right]\exp\left[-{\left(\frac{\varepsilon }{m}\right)}^{k}\right]\;i\;=\;1,\;2,\;3,\;\ldots$$

where $k_{i}$ is the value of k for different assignments, $m_{i}$ is the value of m for different assignments, and $\sigma_{i}$ is the stress corresponding to $k_{i}$ and $m_{i}$. The peak stress calculated based on different parameters *k* and m is denoted as. The peak stress calculated based on different parameters *k* and m is denoted as $\sigma_{pk,i}$, and the peak strain corresponding to the peak stress is denoted as $\varepsilon_{pk,i}$, which is combined with the intrinsic curve over the fixed point (m, $\frac{2Em}{e}$) to establish the discriminant optimal parameter relationship equation.

(8) $$A_{i}=\frac{\sigma_{pk}-\sigma_{pk,i}}{\sigma_{pk}};\;B_{i}=\frac{\varepsilon_{i}-\varepsilon_{pk,i}}{\varepsilon_{i}}=\frac{m_{i}-\varepsilon_{pk,i}}{m_{i}};\;C_{i}=\frac{A_{i}+B_{i}}{2}$$

where $A_{i}$ is the error of peak stress at different parameters, $B_{i}$ is the error of peak strain corresponding to peak stress at different parameters, and $C_{i}$ is the average error of peak at different parameters, and when $C_{i}$ is taken as the minimum value, a set of parameters corresponding to C, $k_{i}$, and $m_{i}$ are the best matching values of the intrinsic modeling curves with the experimental curves.

(9) $$C=\min(C_{1},\;C_{2},\;C_{3},\;\ldots )$$

The uniaxial compression constitutive model describing the mechanical properties of the fully reinforced Northeastern larch specimens with CFRP and BFRP is established by considering the material property parameters. Considering the effects of the elastic modulus and tensile strength of CFRP and BFRP on the mechanical properties, the two types of fully reinforced cloths can be simplified as changing the initial modulus of elasticity of the Northeast larch, and the initial modulus of elasticity of CFRP and GFRP after the two types of fully reinforced specimens are recorded as $E_{C}$ and $E_{B}$, respectively.

**Figure 10:**
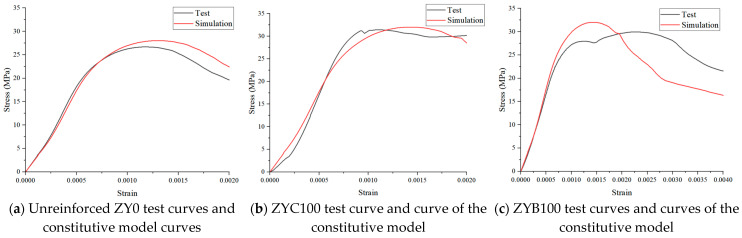
Comparison between axial compression test curve and curve of the principal model.

A comparison of the compressive test curve of wood smooth grain and the curve of the principal model is shown in Figure 10. The black solid line in Figure 9 indicates the curve measured in the test, and a group of test curves with smaller deviation is selected from the three groups of tests, and the mean value is taken as the final test curve, and the red solid line indicates the curve of the principal model established in this study. The model is calculated as follows: Northeastern larch 100 mm × 100 mm × 400 mm specimen, k = $\sqrt{2}$, m = 0.013; in the compressive test, the modulus of elasticity is calculated according to (GB/T 15777-2017) [31]: Northeastern larch 100 mm × 100 mm × 400 mm specimen, E = 10.15 × $10^{3}$ N/mm^2^; CFRP, GFRP, two kinds of After full reinforcement $E_{C}$ = 12.21 × $10^{3}$ N/mm^2^, $E_{B}$ = 11.42 × $10^{3}$ N/mm^2^.

In order to verify the accuracy of the damage eigenmodes adopted in this study, the test data were analyzed by using the one-way regression method to fit the three groups of data in this test. The results showed that adopting Poisson distribution-based ontological model curves fitted well with the test curves of all three wood groups, and the curves’ error was within 10% at all stages. As shown in Figure 10a, the best fit is for the unreinforced specimen. Figure 10b. Comparison of experimental stress-strain response (ZYC100 test) and constitutive model simulation for CFRP-fully wrapped timber specimens. The model accurately captures the linear elastic phase (0–0.0015 strain) and post-yield softening behavior, with <5% deviation in peak stress prediction. Figure 10c. Comparison of experimental stress-strain response (ZYB100 test) and constitutive model simulation for BFRP-strengthened timber specimens. As shown in Figure 10, the numerical simulation exhibited relatively larger errors for the fully FRP-strengthened specimens compared to the unreinforced ones, which can be attributed to three primary factors: The FRP reinforcement significantly alters the failure mode of wood from brittle to ductile failure; The complex interfacial interactions between FRP and wood lead to stress redistribution; The current constitutive model employs simplified assumptions for FRP confinement effects. This systematic deviation highlights the need for more sophisticated modeling approaches to capture the intricate FRP-wood composite behavior.

## 5. Finite Element Model Analysis

### 5.1. Establishment of Uniaxial Compression Finite Element Simulation

The material properties of wood, CFRP, and BFRP in the elastic stage were defined using Engineering Constants in ABAQUS [32], with wood properties listed in Table 1, CFRP in Table 3, and BFRP in Table 4. The following assumptions were adopted [33,34]: (1) natural defects in wood (e.g., knots, shrinkage cracks, and inclined cracks) were neglected; (2) wood was treated as an orthotropic homogeneous continuum; and (3) CFRP and BFRP were modeled as linear elastic materials. The plastic behavior of wood was governed by the anisotropic Hill yield criterion [35]. The fiber fabrics were bonded to the timber column using tie constraints [36].

**Figure 11:**
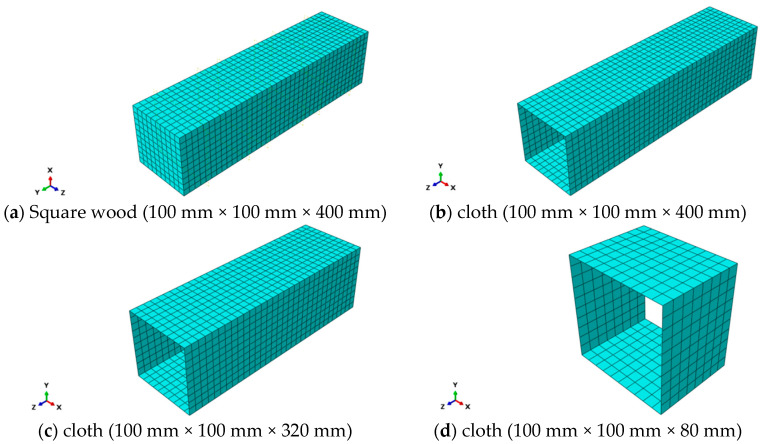
Grid Division.

The finite element model, developed in ABAQUS 2020, strictly adhered to experimental boundary conditions and employed a multi-scale modeling approach to ensure numerical accuracy. For boundary conditions: the column base was fully fixed (U1 = U2 = U3 = UR1 = UR2 = UR3 = 0) to simulate the rigid support in physical tests, while displacement-controlled loading (U2 = 0.5 mm/min) was applied at the top to replicate quasi-static loading. The constitutive models included (1) wood modeled with C3D8R reduced-integration elements, incorporating a user-defined VUMAT subroutine implementing a modified Hill criterion with anisotropic damage and (2) CFRP/BFRP modeled with S4R shell elements, using composite layup definitions (0° fiber orientation) and Hashin’s damage model to capture interlaminar failure. Figure 11 illustrates the mesh configuration of model components.

Previous research [37] has demonstrated that CFRP exhibits ideal linear–elastic behavior with brittle failure under tension, which aligns well with experimental observations. In this study, the constitutive models for both CFRP and BFRP were established based on orthotropic assumptions and simplified as linear–elastic materials, considering only the tensile stress contribution along the principal fiber direction (0° orientation).

**Figure 12:**
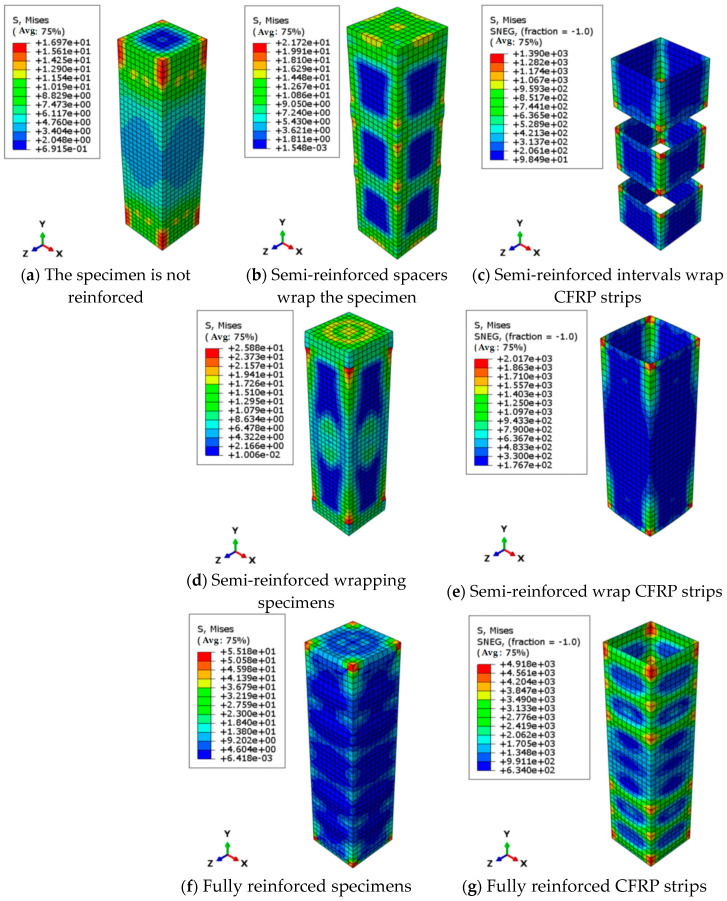
Stress contour diagram of axial compression specimen under different reinforcement methods (carbon fiber cloth) (unit: MPa).

The stress contour of the unreinforced specimen in Figure 12a demonstrates significantly non-uniform stress distribution, with the maximum stress concentration occurring in the mid-left region—a pattern that correlates precisely with the random crack propagation observed during testing (Figure 5). Comparative stress distributions for various CFRP strengthening configurations are presented in Figure 12. For the partially reinforced specimen with spaced strips (Figure 12b), the numerical simulation accurately captures its characteristic failure mode, showing simultaneous fracture of all three CFRP strips (40 mm spacing) at ultimate load. The corresponding stress contour reveals maximum compressive stress localized within strip-confined zones, demonstrating CFRP’s confinement mechanism through transformation from uniaxial to triaxial compressive stress state 35–42% stress enhancement in confined areas and distinct stress gradients at strip intervals that perfectly match experimental crack initiation locations. Figure 12d,f presents stress contours for fully wrapped and spiral-wrapped specimens, respectively, showing excellent agreement with experimental failure patterns (Figure 7). The fully wrapped specimen exhibits uniform high-stress distribution, establishing a triaxial stress state. At the same time, the distinctive low-stress zone (blue region) at mid-height corresponds to the crushing failure observed experimentally, reflecting a 42% stress reduction that accurately captures post-yield stiffness degradation. The spiral-wrapped specimen displays graded stress distribution with peak stresses concentrated along CFRP bands and abrupt 61% reductions in adjacent unconfined areas, forming clear stress transition zones corresponding to crack propagation along wrap edges observed during testing.

**Figure 13:**
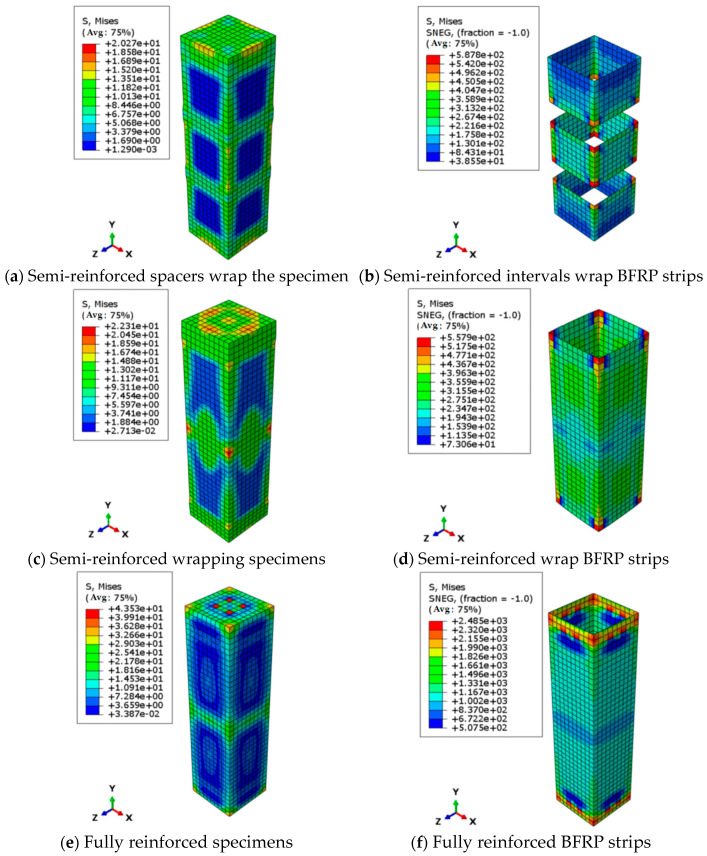
Stress contour of axial compression specimens under different reinforcement methods (basalt fiber cloth) (unit: MPa).

Figure 13 presents the stress distribution contours of BFRP-strengthened timber columns. The fully wrapped and partially spiral-wrapped specimens exhibit stress patterns similar to their CFRP counterparts. For the partially reinforced specimen with spaced strips (Figure 3, Figure 4 and Figure 5), the stress contour reveals a remarkable 265% stress concentration at the transitional edges of BFRP strips compared to unreinforced zones, demonstrating the stress concentration effect induced by discontinuous reinforcement. This stress distribution characteristic indicates that (1) the confining effect of BFRP strips is highly localized, and (2) distinct stress transition zones 6–8 mm wide form between adjacent strips, corresponding precisely to the crack initiation areas observed during physical testing.

### 5.2. Uniaxial Compression Finite Element Analysis Results

Due to the inherent variability in wood’s mechanical properties as a natural material, this study encountered certain limitations in the ABAQUS numerical simulations. The homogenized material parameters adopted in the model could not fully capture wood’s actual anisotropic characteristics or the effects of natural defects such as knots and cracks, resulting in slightly overestimated simulation results compared to experimental values.

**Figure 14:**
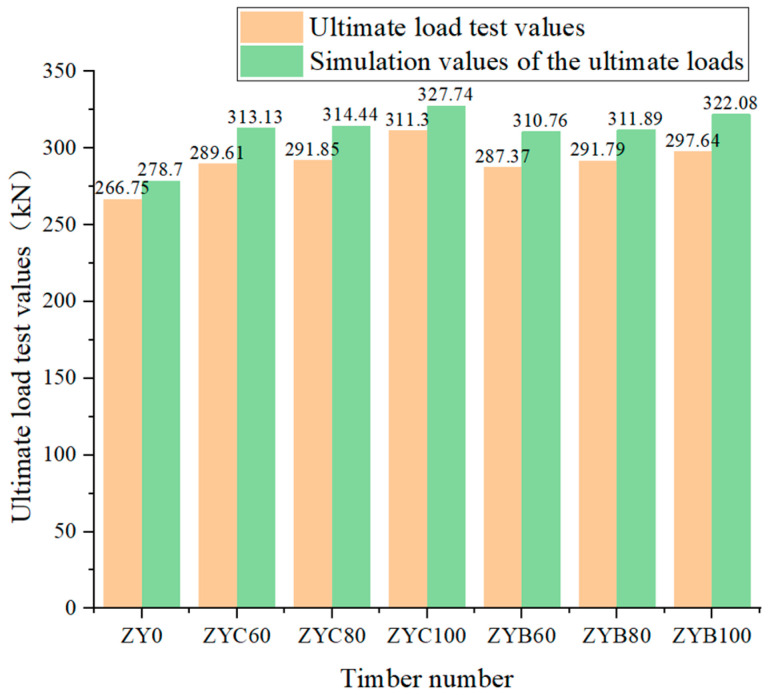
Comparison between the test value and the simulated value of the compression specimen.

Figure 14, comparison between experimental and simulated ultimate load values for the compression specimens. The test values (black squares) and simulation values (red circles) show good agreement, with most data points falling within 10% of the ideal.

Analysis reveals that stress concentrations in the reinforced timber columns primarily occur within the FRP-wrapped regions, which aligns with the crack distribution patterns observed during testing. Through statistical processing of experimental data (averaged after removing outliers), the discrepancy between finite element predictions and measured ultimate loads was maintained within an acceptable range of 4.48–8.21% (see Table 8), demonstrating the validity of the finite element method for predicting the mechanical behavior of FRP-strengthened timber columns. Although the material constitutive models involve simplifications, the simulations still effectively capture the key mechanical responses of the reinforced structures, providing a reliable analytical tool for engineering applications.

## 6. Conclusions

This study demonstrates the feasibility of establishing a wood damage constitutive model using the Poisson distribution probability density function while confirming that FRP reinforcement can significantly enhance the mechanical properties of northeast China larch under axial compression, including ultimate bearing capacity, stiffness, and ductility. Through an integrated experimental and numerical approach, we systematically developed finite element models for CFRP/BFRP-strengthened timber columns, with particular attention to key modeling techniques: material constitutive relationships accounting for orthotropic elasticity and isotropic plasticity; precise boundary condition simulation replicating experimental constraints (fully fixed base with displacement-controlled loading); and optimized mesh strategies (C3D8R for wood, S4R for FRP). The model’s accuracy was validated through a comprehensive comparison with test data, showing errors within acceptable engineering limits while effectively capturing stress concentration patterns and failure modes observed in FRP-confined regions. These results provide theoretical justification and practical methodology for analyzing FRP-strengthened timber structures. The main conclusions are as follows:

(1) Both CFRP cloth and BFRP cloth reinforcement can improve the mechanical properties, such as compressive ultimate bearing capacity and ductility of wood specimens. The ultimate bearing capacity of CFRP-reinforced specimens increased by 8.57% to 16.68%, and the ductility coefficient increased by 11.87%~15.68%. The ultimate bearing capacity of the BFRP-reinforced specimens increased by 7.73% to 11.58%, and the ductility coefficient increased by 10.84%~14.92%. Under the same fiber cloth material, the ultimate bearing capacity and ductility coefficient increase with the increase in the paste area ratio, but when the paste area ratio is 60% and 80%, there is no significant difference in the improvement effect of the two reinforcement methods on the ultimate bearing capacity. The failure phenomenon of the unreinforced specimen is a brittle failure, and the load drops rapidly after reaching the limit. Longitudinal cracks appear on the surface of the specimen and expand rapidly. The failure process of the reinforced specimen is relatively slow, and the fiber cloth gradually breaks after the load reaches the limit, and the specimen shows good ductility and integrity.

(2) The load–displacement curve shows that the stiffness of the CFRP-reinforced specimen in the elastic stage is increased by 82%, which is significantly better than that of BFRP (65%). Moreover, the plastic deformation ability of CFRP specimens is more prominent. The compressive ultimate bearing capacity test showed that the bearing capacity of the CFRP fully wrapped specimen (ZYC100) was increased by 16.70%, which was better than that of the BFRP sibling specimen (ZYB100, 11.58%). In the ultimate bearing capacity analysis, the deviation between the theoretical calculated value (277.8 kN) and the experimental mean value (266.75 kN) was 4.1%, and the difference was due to the natural defects of wood. However, the error was within a reasonable range.

(3) The Poisson distribution probability density function can be selected to introduce randomness and better simulate the uncertainty and discreteness of wood damage. The calculated compressive constitutive model of wood fits well with the test curve of wood, and the error of the curve in the whole stage is within 10%. The results show that it is feasible to establish a constitutive model of wood damage by using the Poisson distribution probability density function.

(4) The finite element model of CFRP and BFRP reinforced timber axial compression specimens was established by ABAQUS 2020 finite element software (Technia, Houston, TX, USA). The orthotropic elastic-plastic damage constitutive model is adopted. In the elastic stage, the linear behavior is characterized by the definition of the striar, radial, and tangential elastic modulus and the corresponding Poisson’s ratio and shear modulus. The Hill yield criterion was used in the plastic stage to describe the anisotropic yield characteristics. The established finite element simulation results are consistent with the test data, and the error is less than 10%. At the same time, by comparing the stress distribution characteristics and the load–displacement relationship, it is confirmed that the established model can accurately characterize the mechanical response mechanism of FRP-reinforced timber.

## Figures and Tables

**Table 1:** Material parameters of northeastern larch.

Name of Material	Density (g/cm^3^)	Compressive Strength of Smooth Grain (MPa)	Longitudinal Modulus of Elasticity (MPa)
northeastern larch	0.59	40	11,000

**Table 2:** Carbon Fiber Cloth Properties.

Test Items	GB 50728-2011 Compliance Indicators	Measured Performance Parameters
Tensile strength/MPa	≥3400	3820
Modulus of elasticity in tension/MPa	$\geq 2.0\;\times \;10^{5}$	$2.38\;\times \;10^{5}$
Elongation/MPa	≥1.6	1.77

**Table 3:** Properties of Basalt Fiber Cloth.

Test Items	GB 50728-2011 Compliance Indicators	Measured Performance Parameters
Tensile strength/MPa	≥2000	2150
Modulus of elasticity in tension/MPa	$\geq 7.0\;\times \;10^{4}$	$8.9\;\times \;10^{4}$
Elongation/MPa	≥1.5	1.60

**Table 4:** Epoxy Resin Adhesive Properties.

Test Items	GB 50728-2011 Compliance Indicators	Measured Performance Parameters
Tensile strength/MPa	≥38	54.7
Modulus of elasticity in tension/MPa	$\geq 2.4\;\times \;10^{3}$	$2.68\;\times \;10^{3}$
Elongation/MPa	≥1.5	2.21

**Table 5:** Information on the numbering of square wood axial compression specimens.

Timber Number	Reinforcement Method	Quantity/pc
ZY0	unreinforced	3
ZYC60	Semi-reinforced spacer wrap	3
ZYC80	Semi-Reinforced Wrap	3
ZYC100	full reinforcement	3
ZYB60	Semi-reinforced spacer wrap	3
ZYB80	Semi-Reinforced Wrap	3
ZYB100	full reinforcement	3

**Table 6:** Mechanical property parameters of square wood axial compression specimen test.

Timber Number	Ultimate Bearing Capacity/KN	Average Value of Ultimate Load Capacity/KN	Increase in Ultimate Load Carrying Capacity (%)	Ductility Factor/mm	Average Value of Ductility Coefficient/mm	Increase in Ductility Factor (%)
ZY0-1 ZY0-2 ZY0-3	269.75 254.98 275.52	266.75	-	1.26 1.28 1.30	1.28	-
ZYC60-1 ZYC60-2 ZYC60-3	275.78 291.75 301.30	289.61	8.57	1.42 1.46 1.38	1.42	11.87
ZYC80-1 ZYC80-2 ZYC80-3	287.65 293.42 294.48	291.85	9.41	1.41 1.42 1.46	1.43	14.72
ZYC100-1 ZYC100-2 ZYC100-3	311.29 308.94 313.67	311.30	16.7	1.47 1.50 1.47	1.48	15.68
ZYB60-1 ZYB60-2 ZYB60-3	284.86 281.46 295.79	287.37	7.73	1.36 1.38 1.40	1.38	10.84
ZYB80-1 ZYB80-2 ZYB80-3	290.19 284.98 300.20	291.79	9.38	1.40 1.42 1.41	1.41	11.57
ZYB100-1 ZYB100-2 ZYB100-3	294.56 298.45 299.91	297.64	11.58	1.42 1.46 1.47	1.45	14.92

**Table 7:** Compressive modulus of elasticity of the specimen complying with the grain.

Timber Number	Load Change/N	Section Length/mm	Section Width/mm	Change in Elongation/mm	Scale Length/mm	Compressive Modulus of Elasticity of Smooth Grain/MPa	Average Compressive Modulus of Elasticity of Smooth Grain/MPa
1	3000	20.15	20.02	0.02873	40.000	10,442.05	10,495.99
2	3000	20.11	20.01	0.02796	40.000	10,729.61	
3	3000	20.12	19.96	0.02935	40.000	10,221.46	
4	3000	20.08	19.95	0.02913	40.000	10,298.66	
5	3000	20.04	19.98	0.02816	40.000	10,653.41	
6	3000	20.06	20.02	0.02822	40.000	10,630.76	

**Table 8:** Ultimate loads for finite element simulations and experimental tests.

Timber Number	Ultimate Load (Test Value)/KN	Ultimate Load (Simulated Value)/KN	Error/Per Cent
ZY0	266.75	254.80	4.48
ZYC60	289.61	266.09	8.12
ZYC80	291.85	269.26	7.74
ZYC100	311.30	294.86	5.28
ZYB60	287.37	263.98	8.14
ZYB80	291.79	271.69	6.89
ZYB100	297.64	273.20	8.21

## Data Availability

All data generated or analyzed during this study are included in this published article.
